# Feasibility to use whole-genome sequencing as a sole diagnostic method to detect genomic aberrations in pediatric B-cell acute lymphoblastic leukemia

**DOI:** 10.3389/fonc.2023.1217712

**Published:** 2023-08-14

**Authors:** Fatemah Rezayee, Jesper Eisfeldt, Aron Skaftason, Ingegerd Öfverholm, Shumaila Sayyab, Ann Christine Syvänen, Khurram Maqbool, Henrik Lilljebjörn, Bertil Johansson, Linda Olsson-Arvidsson, Christina Orsmark Pietras, Anna Staffas, Lars Palmqvist, Thoas Fioretos, Lucia Cavelier, Linda Fogelstrand, Jessica Nordlund, Valtteri Wirta, Richard Rosenquist, Gisela Barbany

**Affiliations:** ^1^ Department of Molecular Medicine and Surgery, Karolinska Institutet, Stockholm, Sweden; ^2^ Department of Clinical Genetics, Karolinska University Hospital, Stockholm, Sweden; ^3^ Science for Life Laboratory, Karolinska Institutet, Stockholm, Sweden; ^4^ Department of Medical Sciences, Uppsala University, Uppsala, Sweden; ^5^ Science for Life Laboratory, Uppsala University, Uppsala, Sweden; ^6^ Science for Life Laboratory, Department of Microbiology, Tumor and Cell Biology, Karolinska Institutet, Stockholm, Sweden; ^7^ Division of Clinical Genetics, Department of Laboratory Medicine, Lund University, Lund, Sweden; ^8^ Department of Clinical Genetics, Pathology, and Molecular Diagnostics, Office for Medical Services, Region Skåne, Lund, Sweden; ^9^ Department of Clinical Genetics and Genomics, Sahlgrenska University Hospital, Gothenburg, Sweden; ^10^ Department of Microbiology and Immunology, Institute of Biomedicine, Sahlgrenska Academy, University of Gothenburg, Gothenburg, Sweden; ^11^ Department of Clinical Chemistry, Sahlgrenska University Hospital, Gothenburg, Sweden; ^12^ Department of Laboratory Medicine, Sahlgrenska Academy, University of Gothenburg, Gothenburg, Sweden; ^13^ Clinical Genomics Lund, Science for Life Laboratory, Lund University, Lund, Sweden; ^14^ Science for Life Laboratory, School of Engineering Sciences in Chemistry, Biotechnology and Health, KTH Royal Institute of Technology, Stockholm, Sweden; ^15^ Genomic Medicine Center Karolinska, Karolinska University Hospital, Stockholm, Sweden

**Keywords:** B-cell acute lymphoblastic leukemia, whole-genome sequencing, genomic aberrations, diagnostic validation, class-defining genetic lesions

## Abstract

**Introduction:**

The suitability of whole-genome sequencing (WGS) as the sole method to detect clinically relevant genomic aberrations in B-cell acute lymphoblastic leukemia (ALL) was investigated with the aim of replacing current diagnostic methods.

**Methods:**

For this purpose, we assessed the analytical performance of 150 bp paired-end WGS (90x leukemia/30x germline). A set of 88 retrospective B-cell ALL samples were selected to represent established ALL subgroups as well as ALL lacking stratifying markers by standard-of-care (SoC), so-called B-other ALL.

**Results:**

Both the analysis of paired leukemia/germline (L/N)(n=64) as well as leukemia-only (L-only)(n=88) detected all types of aberrations mandatory in the current ALLTogether trial protocol, i.e., aneuploidies, structural variants, and focal copy-number aberrations. Moreover, comparison to SoC revealed 100% concordance and that all patients had been assigned to the correct genetic subgroup using both approaches. Notably, WGS could allocate 35 out of 39 B-other ALL samples to one of the emerging genetic subgroups considered in the most recent classifications of ALL. We further investigated the impact of high (90x; n=58) vs low (30x; n=30) coverage on the diagnostic yield and observed an equally perfect concordance with SoC; low coverage detected all relevant lesions.

**Discussion:**

The filtration of the WGS findings with a short list of genes recurrently rearranged in ALL was instrumental to extract the clinically relevant information efficiently. Nonetheless, the detection of *DUX4* rearrangements required an additional customized analysis, due to multiple copies of this gene embedded in the highly repetitive D4Z4 region. We conclude that the diagnostic performance of WGS as the standalone method was remarkable and allowed detection of all clinically relevant genomic events in the diagnostic setting of B-cell ALL.

## Introduction

Genetic characterization of acute lymphoblastic leukemia (ALL) is mandatory in modern treatment protocols since it provides important prognostic information, which, together with measurements of initial treatment response, is used to adjust treatment intensity within risk-adapted protocols (1–3). The genomic landscape of ALL is very heterogeneous and extends from aneuploidies over structural variants (SVs) to focal copy number alterations (CNAs) and single-nucleotide variants (SNVs) (4, 5). To accurately detect this range of aberrations, the diagnostic work-up requires a multimodal procedure, combining screening and targeted methods, which makes SoC genetic diagnostics cumbersome and labor-intensive (4–6). Still, a significant proportion of both pediatric and adult ALL patients lack recognized genetic markers, and for these patients, the genetic findings do not contribute to risk stratification.

In the past decade, high-throughput sequencing technologies have provided new tools to unravel the genomics of ALL and led to the identification of novel genomic aberrations with potential prognostic impact or implications for targeted therapy (7). Based on these new findings, the latest WHO 2022 classification (8) and the International Consensus Classification (ICC) (9) propose a number of emerging ALL subgroups among B-cell ALL, significantly decreasing the number of patients lacking primary genetic lesion, the so-called B-other ALL. While these novel aberrations are not yet mandatory to investigate in contemporary treatment protocols, these genetic lesions have been suggested to impact outcomes and may thereby contribute valuable information to patient management (10–16).

Recent studies have shown a high accuracy and cost-efficiency of whole-genome sequencing (WGS) in the diagnostic setting of germline conditions (17) as well as cancer (18). In hematological malignancies specifically, WGS was recently demonstrated to be superior to conventional methods, adding clinically relevant information in 25% of patients with myelodysplastic syndrome or acute myeloid leukemia, information that changed patient stratification for 16% of the patients (19). Also, in B-cell ALL, a recent report by Ryan and coworkers showed that WGS displayed a high diagnostic yield, as subtype-defining lesions were detected in 97% of patients. Of note, restricted analysis of leukemic samples (L-only) could call primary genetic abnormalities in 37 out of 38 patients (20). A further study based on WGS investigated a subset of pediatric B-other ALL patients from the UKALL2003 trial and identified a subtype-defining lesion in 94% of the patients (15), whereas a similar study of 47 adult B-other ALL patients detected a class-defining aberration in 87% of patients (21).

The performance of WGS in the diagnostic setting of hematological malignancies is thus far very promising; however, there are still technical and interpretation issues that need to be addressed before current methods can be replaced by WGS. In the present study, we assessed the diagnostic yield and accuracy of WGS as the sole diagnostic method to detect genetic lesions of clinical relevance in the diagnostic setting of ALL. To this end, we challenged WGS and the bioinformatics pipeline to identify clinically relevant genetic aberrations in a set of 88 well-characterized retrospective B-cell ALL cases. We applied sequential filtration to extract the clinically relevant information efficiently and assessed the accuracy through the comparison to SoC findings. Furthermore, we evaluated the sensitivity of high (90×) versus low (30×) coverage and compared the diagnostic yield of paired leukemia-germline samples to L-only analysis, as these parameters strongly influence turnaround time and costs. The overall results showed a complete concordance between SoC and WGS, also when restricting the analysis to L-only, and that WGS could assign the majority of B-other ALL to emerging genetic subgroups.

## Materials and methods

### Patient samples

We investigated 88 retrospective leukemic bone marrow (BM) samples from patients diagnosed with pediatric B-cell ALL, treated according to NOPHO trials protocol at Uppsala University Hospital and Karolinska University Hospital in Sweden. The cases were selected to represent the mandatory genetic subgroups (**Supplementary Table 1**) defined in the current protocol (n = 49), and the remaining samples consisted of B-cell ALL cases lacking recognized stratifying aberrations, i.e., the B-other group (n = 36), as well as three patients with Down syndrome ALL (DS-ALL). The blast count was above 50% in all but two samples: P111 with an *ETV6*::*RUNX1* fusion present in 37% of the cells and P120 with a *KMT2A* rearrangement (*KMT2A*-r) present in 14% of the cells. The distribution of B-cell ALL samples across genetic subgroups is summarized in **Table 1**. The samples were divided into an exploratory set (n = 58) and a validation set (n = 30). BM samples taken during follow-up, when the patients were in remission, were used as the source of germline DNA (n = 64) for the paired analysis.

**Table 1:** Summary of samples analyzed.

Aberrations	L 90×/N 30×	L 30×	Sum
Low hypodiploidy	–	1	1
High hyperdiploidy	11	3	14
t(12;21)	7	2	9
t(1;19)	3	2	5
iAMP(21)	5	1	6
t(9;22)	3	1	4
*KMT2A*-r	6	1	7
ABL-class	3		3
B-other	27/23	9	36
DS-ALL	3	–	3
Sum	68/64	20	88

t(12;21), translocation between chromosomes 12 and 21; t(1;19), translocation of chromosomes 1 and 19; iAMP(21), intrachromosomal amplification of chromosome 21; t(9;22), translocation of chromosomes 9 and 22; *KMT2A*-r, *KMT2A* rearrangements; DS-ALL, Down syndrome acute lymphoblastic leukemia; L, leukemia only; N, paired germline sample; 90×/30×, sequence depth.

At diagnosis, all samples were genetically characterized according to SoC as specified in the NOPHO protocols, which included chromosome banding analysis, fluorescence *in situ* hybridization (FISH) analysis to investigate the presence of t(12;21), t(9;22), *KMT2A*-r, intrachromosomal amplification of chromosome 21 (iAMP (21)) (Abbott, Vysis, Abbott Park, IL, USA), t(1;19) (MetaSystems Probes, Heidelberg, Germany), and dic(9;20) (Kreatech Diagnostics, Amsterdam, Netherlands) as well as array comparative genomic hybridization/single-nucleotide polymorphism (CGH/SNP) array analysis. For the purpose of the study, the genetic subgroup for the samples from the NOPHO 92 and 2000 trials (n = 22) was updated to SoC in the NOPHO 2008 trial, as the older trials investigated fewer aberrations (22, 23). Also, three of the B-other samples that harbored retrospectively detected *ABL*-class rearrangements were revised, as this subgroup was not investigated in the NOPHO 2008 trial.

### Sample preparation

BM samples were collected in 5-ml EDTA tubes, and genomic DNA (gDNA) was isolated using a Tissue kit an EZ1™ automated instrument (Qiagen, Hilden, Germany) or extracted manually from frozen cell pellets with the AllPrep DNA/RNA Mini Kit Qiagen (Qiagen, Hilden, Germany). DNA was stored at −20°C until use. The DNA from frozen BM samples taken at remission was extracted with a Gentra Puregene Blood core kit (Qiagen, Hilden, Germany).

### Whole-genome sequencing

For the samples in the exploratory set (n = 58), library preparation and sequencing were performed at the National Genomics Infrastructure, Science for Life Laboratory, Stockholm (n = 36) and Uppsala (n = 22), Sweden. For samples with sufficient amounts of DNA available (36/58), the libraries were prepared using 1 µg of DNA with the TruSeq DNA PCR-free (Illumina, San Diego, CA, USA) protocol, 350-bp insert size, and sequenced using the HiSeq X platform (Illumina), 2 × 150-bp paired-end to ~90× coverage for leukemia samples, and ~30× coverage for the paired germline sample. For samples where little DNA was available (n = 22), 100 ng of input DNA was used for library preparation with TruSeq DNA Nano protocol (Illumina).

The samples in the validation set (n = 30) were processed at Clinical Genomics, SciLifeLab, Stockholm. Libraries were prepared using the NxSeq^®^ AmpFREE Low DNA Library Kit (Lucigen, Biosearch Technologies, Petaluma, CA, USA) with 200 ng of DNA input and thereafter sequenced on the NovaSeq 6000 instrument (Illumina) using paired-end 150-bp reads to ~90× (n = 10) or ~30× coverage (n = 20).

To investigate how sequencing depth influenced variant detection, 10 leukemia samples (originally sequenced to 90× coverage) were down-sampled *in silico* to a coverage of approximately 30×. Down-sampling was performed by randomly discarding reads and retaining only every third read. The subsequent downstream analysis was performed in an identical manner to the 90× L-only analysis.

### Data processing

The sequence data were converted to FASTQ format using Illumina bclfastq and further processed using Sarek v2.5.1 (24), a Nextflow pipeline (25) from the nf-core framework of community-curated bioinformatics pipelines (26) on the UPPMAX Cluster (27) at Uppsala University. Briefly, preprocessed FASTQ files were checked for quality with FASTQC (28) before being aligned with BWA-mem (29) to the human reference genome build GRCh37/hg19. Duplicates were marked with Picard MarkDuplicates (30) before base calibration and indel realignment with GATK tools were performed (30), and quality statistics were aggregated with the help of multiqc (31). Following the pre-processing steps, Bam files were generated.

Sequencing data for the validation set were processed at Clinical Genomics through BALSAMIC (Bioinformatic Analysis pipeline for Somatic Mutations in Cancer) version 10.0.5, which packages the workflows for variant calling indicated below (32). Subsequently, the variant files were uploaded to the VCF visualization interface, SCOUT (33), available at https://github.com/Clinical-Genomics/scout, for further inspection and interpretation.

### Detection of aneuploidies, copy-number alterations, and single-nucleotide variants

The vcf2cytosure v0.7.1, included in the BALSAMIC v10.0.5 converter (34) was applied to visualize ploidy changes and CNAs (35). The tool converts the output from the variant calling to a “.cgh” format used by CytoSure™ Interpret Software (Oxford Gene Technologies, Oxford, UK) originally developed to display oligonucleotide microarray measurements. Briefly, output files were binned, and 20 bins were pooled into one probe. Coverage along the genome was calculated as the log2 ratio for individual bins relative to all bins. The mean coverage in the sample was drawn at height 0 and regarded as the log2 ratio for the diploid genome (n = 2); ratios above this threshold indicate gains, and below losses. As the tool calculates the threshold relative to the average coverage over the entire genome, this threshold was adjusted manually for samples with multiple tri/tetrasomies or monosomies.

We also used allele-specific copy-number analysis of the leukemia samples (ASCAT version 4.5.0) (36), also included in BALSAMIC, to visualize aneuploidies and copy-number neutral loss of heterozygosity (CNN-LOH), with the latter requiring a paired germline sample.

Additionally, two recurrent SNVs, *PAX5* P80R and *IKZF1* N159Y (9), were also assessed in samples analyzed through the clinical platform.

### Detection of structural variation

SV calling was conducted using the FindSV (37), which merges the variants callers CNVnator (38) and TIDDIT (39), and the variant effect predictor (VEP) (40) was subsequently applied. BALSAMIC version 10.0.5 uses Manta version 1.6.0 (41), Delly version 1.0.3 (42), and TIDDIT version 3.0.0 (31) to call SVs. Subsequently, the output files were annotated in SWEGEN, a reference cohort, that reflects the genetic structure of the Swedish population (43), and variants with an allele frequency above the threshold of 0.02 were discarded. In addition to SWEGEN, the samples in the validation set were also annotated in locusDB, a locally curated database with non-tumor samples, and variants with an observed frequency above 0.02 were discarded as well (44). The SV workflow detected a median of 21,500 SVs per sample, which decreased to 7,500 once recurrent variants above the 0.02 threshold were removed (and further down to roughly 4,000 when variants in the paired germline were subtracted) (leukemia/normal, L/N). Finally, a short list of clinically relevant genes (**Supplementary Table 2**) was applied, and the filtered events were inspected manually in IGV. The L-only analysis was carried out in the same manner, omitting the filtration with the paired normal.

### Detection of *DUX4* rearrangements

The location of *DUX4*, within the highly repetitive D4Z4 region (45), results in a high number of copies for *DUX4* and *DUX4*-like genes. This poses challenges for the detection of *DUX4*-r with short-read WGS. To overcome the limitations of our initial bioinformatics pipeline, we applied a SAMtools command (46, 47) to specifically identify reads supporting an *IGH*::*DUX4* rearrangement. The command identifies discordant reads in the *IGH* region (14:106032614–107288051, in GRCh37/hg19), and subsequently, these reads were filtered to only include reads that either i) have a mate mapped to one of the regions where *DUX4* may map (4:190988100–191007000, 10:135477000–135500000, or GL000228.1:7000–115000 in GRCh37/hg19) or ii) have a secondary alignment in either of the above-mentioned regions. See Supplementary Material for the SAMtools command.

### Selection of genes/genomic aberrations included in the shortlist

The aberrations mandatory to investigate according to the current ALL treatment trial protocol (48) were all selected, as were the genes/genomic regions included in the UKALL-CNA classifier and IKZF1+ profiles (49, 50). Furthermore, the key genes signaling genetic subtypes with potential diagnostic or therapeutic implications among B-other ALL (10–16, 51–55) or included in WHO 2022 classification (8) or the ICC of hematological malignancies were also included (9). The genes’ IDs and genomic coordinates are listed in **Supplementary Table 2**.

## Results

### Detection of mandatory aberrations

To investigate whether the detection of all mandatory genetic aberrations was feasible, 38 samples from patients with pediatric B-cell ALL, representative of the genetic subgroups stipulated by the ALLTogether trial protocol (48), were analyzed with the corresponding paired sample (**Table 1**). These included samples with high hyperdiploidy (HeH) and mandatory aberrations caused by SVs, resulting in recurrent fusion genes or iAMP (21). Two different visualization approaches were used to visualize aneuploidies and large CNAs, vcf2cytosure and ASCAT. Both applications allowed for an accurate calling of trisomies/tetrasomies, and the findings were identical to those obtained by SoC for all 11 samples with HeH (**Supplementary Figure 1A**). Equally, the high-risk aberration iAMP (21) was readily identified by both, although inspection to determine the boundaries of the changes along chromosome 21q was only possible in vcf2cytosure (**Supplementary Figure 1B**).

**Figure 1:**
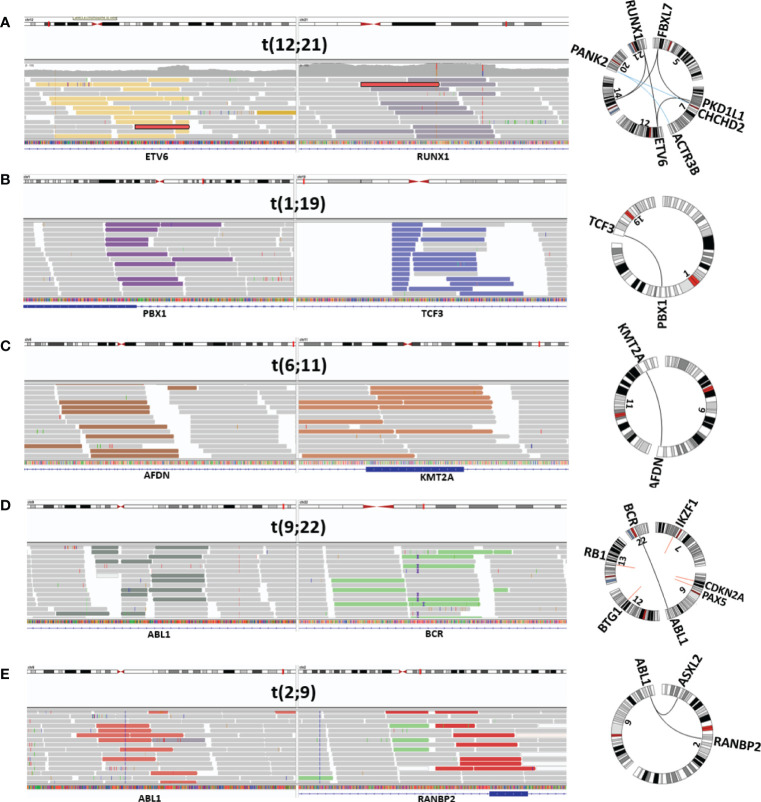
Visualization of mandatory SV. Depiction from IGV and Circos plot illustrating representative mandatory aberrations. The discordant reads at both ends of the junction are displayed in colored bars, and concordant reads are displayed in gray. **(A)** *ETV6::RUNX1-r* (P030), **(B)** *TCF3::PBX1* (P034), **(C)** KMT2A-r (*AFDN::KMT2A)* (P039), **(D)** Philadelphia-positive ALL (*BCR::ABL1*) (P044), and **(E)** ABL-class ALL (*RANBP2::ABL1*) (P047). SV, structural variant; ALL, acute lymphoblastic leukemia.

Filtering SVs with the short list of genes recurrently rearranged in ALL returned 20–25 variants per sample and made manual inspection in IGV feasible. With the use of this approach, all seven samples harboring an *ETV6::RUNX1* fusion were identified, irrespective of whether the fusion arose through a balanced translocation or a complex genomic rearrangement involving multiple chromosomes (**Figure 1A**). Similarly, the *TCF3::PBX1* fusion, resulting from a translocation between chromosome arms 1q and 19p (**Figure 1B**), was identified in all three cases. In the initial analysis, only four out of six *KMT2A-r* with the corresponding fusion partner were detected (**Figure 1C**). Revision of the VCF files revealed that the missing two rearrangements had been discarded in the paired analysis with the germline. Those two follow-up samples had been taken shortly before the patients relapsed, and as the *KMT2A*-r already had reappeared, it had been discarded. t(9;22) was successfully detected in the two Philadelphia-positive ALL samples (**Figure 1D**), both displaying the minor BCR breakpoint and juxtaposition of exon 1 in *BCR* to exon 2 in *ABL1*. Two ABL-class rearrangements involving *PDGFRB* were also identified, whereas a known *RANBP2::ABL1* fusion was missing from the variant list for P047. Manual scrutiny of the variants annotated by VEP in IGV revealed that the *ABL1* breakpoint mapped immediately upstream of the gene. By replacing the gene’s ID with the genomic coordinates and adding 5 kb upstream, the variant was retained in the filtration step, and the *RANBP2::ABL1* fusion was detected (**Figure 1E**).

Next, we assessed whether the mandatory aberrations could be confidently detected through analysis of L-only. Also in this case, filtering with the shortlist narrowed the SVs to manageable numbers and detected all the sought rearrangements, including the *KMT2A*-r that were not detected in the initial L/N analysis. Otherwise, the findings with both approaches were identical, and all the class-defining aberrations were detected. **Supplementary Table 3** lists the findings in the samples with mandatory aberrations by SoC and WGS.

**Figure 2:**
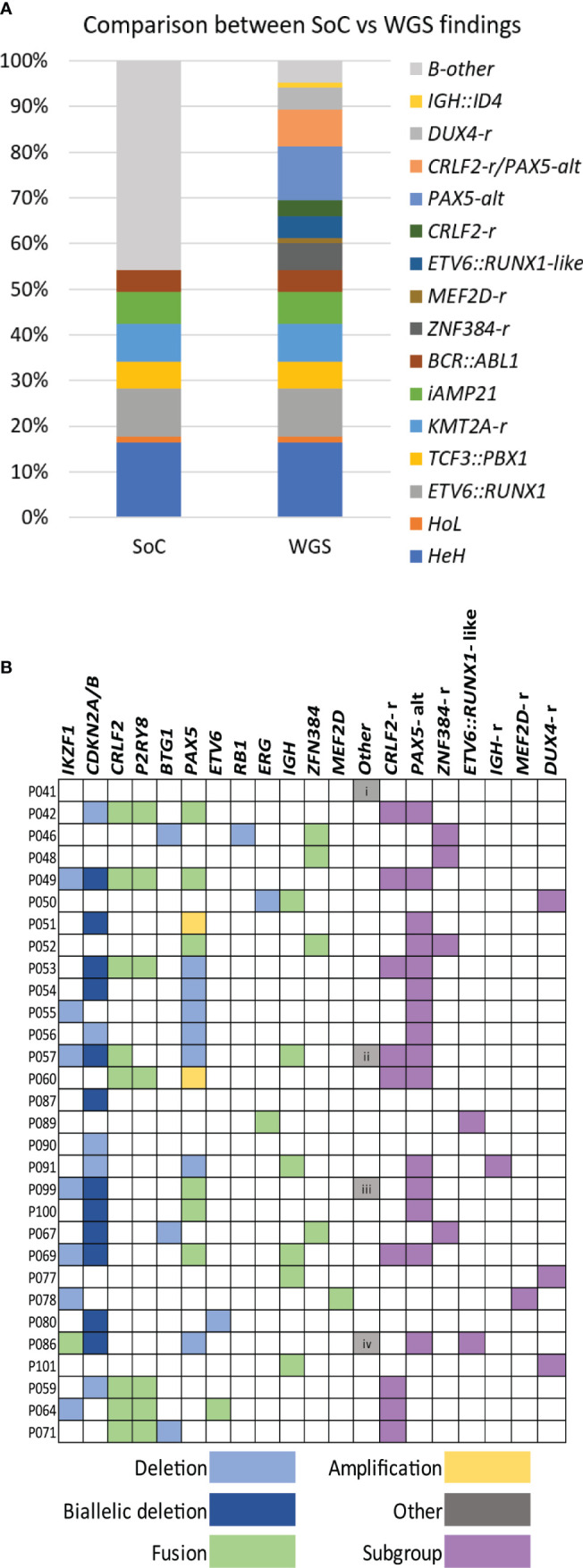
WGS findings. **(A)** Histogram summarizing the class-defining findings by SoC and WGS analysis. **(B)** Distribution of genetic findings in B-other cases is presented in colors. Deletions, amplifications, and fusions are detected and used to assign cases to a genetic subgroup. Other additional findings are i) uniparental disomy (UPD) 14, ii) *KLHL2::MLLT3*, iii) *APBB2::NOL4L*, and iv) *IGK::IKZF1*. WGS, whole-genome sequencing; SoC, standard of care.

To validate the performance in the clinical setting, 10 additional samples (marked with a single asterisk, **Supplementary Table 3** and **Supplementary Table 4**) were processed and analyzed through the clinical platform in a blinded manner. Subsequent comparison with SoC revealed that all relevant variants had been detected by the pipeline through the analysis of paired L/N as well as L-only. Mandatory aberrations were identified in seven samples (**Supplementary Table 3**), two of the remaining samples harbored rearrangements of *PAX5* (P099 and P100), and no single lesion was identified in the third sample (P101). The comparison to SoC showed complete concordance for all 10 cases (**Supplementary Table 4**). In summary, WGS was able to detect all class-defining aberrations that are mandatory to investigate in the current ALL treatment trial with accuracy comparable to SoC. **Figure 2A** illustrates the comparison of the genetic findings by SoC and WGS for the entire set of samples.

### Detection of copy-number alterations in the UKALL-CNA classifier and *IKZF1* ^PLUS^ profile

In addition to SVs and aneuploidies, we investigated whether WGS is suitable to detect CNAs affecting the eight loci included in the UKALL-CNA classifier (49) as well as the *IKZF1* ^PLUS^ profile (56). Deletions of various sizes from single exons to the entire *IKZF1* were detected in vcf2cytosure and also by the SV callers (**Supplementary Figure 2A**), both among the established mandatory subgroups and B-other ALL. CNAs affecting *PAX5*, such as amplification of exons 1–5 (**Supplementary Figure 2B**), intragenic deletions, and partial deletions leading to a *PAX5::ZCCHC7* fusion, were also detected in both groups, albeit at a higher frequency in B-other ALL. The same was true for losses of *CDKN2A* and *CDKN2B*. Deletions in *RB1*, *EBF1*, *ETV6*, *ERG* (**Supplementary Figure 2C**), and *BTG1* were identified in isolated cases, whereas CNAs in the PAR1 region (**Supplementary Figure 2D**), which resulted in the juxtaposition of *P2RY8* and *CRLF2*, were only observed among B-other ALL and DS-ALL. Deletion of *ERG* was the only lesion detected in two of the B-other samples (P050 and P114). Investigation of CNAs was not mandatory in the NOPHO 2008 trial protocol; however, the few samples with available information were concordant with the WGS findings.

### Diagnostic yield in B-other ALL

A total of 36 B-other ALL and three DS-ALL, lacking stratifying aberrations in SoC, were investigated to determine the diagnostic yield. The analysis was restricted to recurrent aberrations that either define subgroups within the B-other group or have been suggested to impact outcome. Among these patients, 35 could be tentatively assigned to one of the emerging genetic categories based on the WGS findings. The lesions identified in B-other ALL are summarized in **Figure 2B** and **Supplementary Table 4**.

The most frequent aberrations were rearrangements that affected *PAX5* (n = 17) and *CRLF2* (n = 10). The alterations in *PAX5* (*PAX5*-alt) were heterogeneous and included recurrent fusions with *NOL4L* (n = 4) (**Supplementary Figure 3A**), *ZCCHC7* (n = 3), *SNTA1* (n = 1), and *DACH2* (n = 1), deletions (n = 9), or amplifications (n = 2) (13). *PAX5*-alt often co-occurred with other putative class-defining lesions, in particular samples harboring *CRLF2* rearrangements (*CRLF2*-r). Both previously described types of *CRLF2-*r were found, i.e., translocations t(X;14)(p22.33;q32) (**Supplementary Figure 3B**) juxtaposing the *IGH* locus to *CRLF2* (n = 3) and a *P2RY8::CRLF2* fusion (n = 7) (57). Interestingly, *CRLF2-*r always co-occurred with *PAX5*-r, with the exception of the three DS-ALL that carried *P2RY8*::*CRLF2* fusions as the only detected lesion. WGS also identified four cases harboring *ZNF384* fusions; the partners were *TCF3* (n = 3) (**Supplementary Figure 3C**) and *TAF15* in P067. An *ETV6*::*IKZF1* fusion signaling *ETV6*::*RUNX1*-like subgroup was detected in P105. Another sample harbored an *ETV6* deletion together with the *KMT2C*::*IKZF1* fusion, and as both lesions associate with *ETV6*::*RUNX1*-like ALL, the sample was classified as such (51). A *FUS*::*ERG* fusion was detected in sample P089. This fusion is rare but recurrent in ALL and has been associated with the *ETV6*::*RUNX1*-like gene expression profile (13). Two recurrent aberrations were also found in one patient each, a *MEF2D*::*BCL9* fusion (P078) and an *IGH::ID4* fusion (P091).

In addition, WGS detected isolated *ERG* deletions in one sample (P050) and a rearrangement affecting the *IGH* locus in sample P077. The corresponding discrepant pairs mapped to the long non-coding RNA *CCDC26* at 8q24; however, no cluster of discordant reads supporting *DUX4*-r was found in our initial analysis, despite extensive manual scrutiny in IGV. In an attempt to overcome this shortcoming, we investigated all samples sequenced to a depth of 90× (n = 68), applying a command that specifically returns the number of discordant reads that link the IGH locus to any of the copies of *DUX4*/*DUX4*-like genes in hg19. This approach was successful and identified clusters of discordant reads in these two samples and sample P101 with no lesion detected previously. As many as 126, 31, and 183 read pairs supported an *IGH::DUX4* rearrangement in P050, P077, and P101, respectively (**Supplementary Table 5**), while the median number of discordant read pairs in the remaining 65 samples was 0 (range, 0–6).

No putative class-defining lesion was identified in the remaining four B-other samples; the only lesions found by WGS were loss of *CDKN2A/B*, uniparental disomy (UPD) for chromosome 14, and *ETV6* deletion (**Figure 2B**; **Supplementary Table 4**).

### Analysis of WGS L-only 30× coverage

Finally, we investigated whether decreasing the coverage to 30× and L-only could be suitable in the diagnostic setting with two approaches. First, we *in silico* (58) down-sampled 10 leukemia samples (marked with two asterisks, **Supplementary Table 3** and **Supplementary Table 4**), containing various aberrations (*ETV6*::*RUNX1*, *TCF3::PBX1*, *KMT2A-r*, *BCR::ABL1*, iAMP (21), and five B-other ALL) to 30× coverage and repeated the analysis described above. The comparison revealed that all the variants detected, i.e., aneuploidies, SVs, and focal CNAs, were also detectable in the corresponding down-sampled 30× data, and all 10 samples were allocated to the correct genetic subgroup.

**Figure 3:**
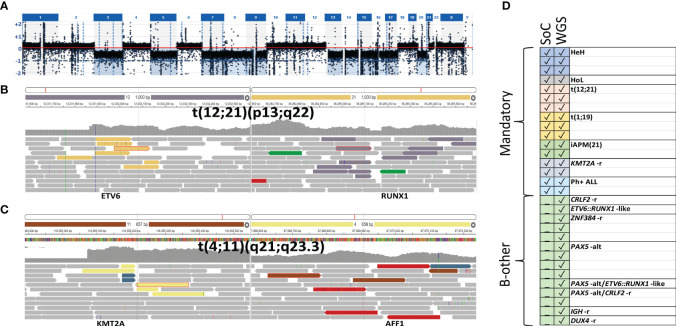
Illustration of representative variants detected through 30× L-only. Depiction from SCOUT’s plug-in IGV showing the discordant reads at both ends of the junction displayed in colored bars and concordant reads displayed in gray. **(A)** Low hypodiploidy (P116) screenshot from vcf2cytosure. The red line is set to indicate the signal intensity corresponding to diploid chromosomes; signals below indicate losses. **(B)** *ETV6::RUNX1* (P111) and **(C)** *KMT2A::AFF1* t(4;11) (P120). **(D)** Summary of the class-defining aberrations found with SoC versus WGS in 10 down-sampled as well as samples sequenced to 30×. SoC, standard of care; WGS, whole-genome sequencing.

Subsequently, DNA from 20 additional diagnostic BM samples, including two samples with low blast counts of 37% and 14%, respectively, was sequenced to 30× coverage and processed as described for L-only samples. A mandatory aberration or putative driver event was identified in 19/20 samples (**Table 2**). Chromosomal gains as in HeH or losses as in HoL (**Figure 3A**), as well as SVs leading to recurrent fusions, were correctly detected. Even in the samples containing a low percentage of blasts, 30× coverage could identify the class-defining lesion, e.g., the *ETV6*::*RUNX1* fusion in P111 (**Figure 3B**) and the *KMT2A::AFF1* fusion P120 (**Figure 3C**). *ERG* deletion was the only somatic aberration identified in sample P114. Targeted analysis revealed 34 read pairs in support of an *IGH*::*DUX4* rearrangement compared to a median of 0 (range 0 to 3) pairs for the rest of the cases, thus confirming the presence of a *DUX4* rearrangement in sample P114. A summary of the findings by SoC and WGS is presented in **Figure 3D**. Taken together, the results indicate that decreasing the sequencing depth to 30× enables the identification of clinically relevant genomic lesions in all the samples investigated irrespective of blast count.

**Table 2:** Summary of SoC and WGS findings in 30× L-only.

UPN	Standard of care karyotype	Additional SoC genetic findings	WGS revised karyotype	WGS additional findings	Subgroup/s SoC/WGS	% blasts at diagnosis
P104	56,XY,+X,+Y,+4,+6,+14,+17,+del(18)(q11.2),+21,+21,+21		Seq[GRCh37] 55,XY,+X,+Y,+4,+6,+14,+17,der(18)?t8;21)(q11.2;q21.2),+21,+21		HeH/HeH	91%
P109	55,XY,+X,+4,+6,8,+10,+14,+17,+18,+21	Deletions *IKZF1* and *EBF1*	Seq[GRCh37] 55,XY,+X,+4,+6,+8,+10,+14,+17,+18,+21	Deletions *IKZF1* and *EBF1*	HeH/HeH	67%
P112	52,XY,+X,+del(4)(q13.3),+9,+14,+21,+21	Deletions *BTG1*, *EBF1*, *IKZF1* (partial) and *RB1*	Seq[GRCh37]52,XY,+X,+del(4)(q13.3),+9,+14,+21,21	Deletions *BTG1*, *RB1*, and *EBF1*	HeH/HeH	50%
P116	35,XX,-3,-5,-7,-8,-9,-13,-14,-15,-16,-17,-20/70,idemx2		Seq[GRCh37] 35,XX,-3,-5,-7,-8,-9,-13,-14,-15,-16,-17,-20		HoL/HoL	78%
P111	46,XY,dup(6)(p12.3p25.3),del(12)(p11.1),del(13)(q14.3).nuc ish(ETV6x1,RUNX1x3)(ETV6 con RUNX1x1)[74/200]		Seq[GRCh37] 46,XY,dup(6)(p25.3p12.3),del(12)(11.1)t(12;21)(p13;q22),del(13)(q14.3)	*ETV6::RUNX1*	*ETV6::RUNX1/ETV6::RUNX1*	37%
P113	46,XY,t(4;6)(q2?1;q2?5),del(12)(p13.3p12.2).nuc ish(ETV6x1,RUNX1x3)(ETV6 con RUNX1x1)[163/200]		Seq[GRCh37] 46,XY,der(4)del(4)(q21.2q22.3), t(4;6)(q21.2;q23.2),der(6)del(6)(q23.3q25.1)t(4;6),del(12)(p13.31p12.2),t(12;21)(p13;q22)	*ETV6::RUNX1* and *PAX5:ZCCHC7*	*ETV6::RUNX1/ETV6::RUNX1*	81%
P110	46,XX,der(19)t(1;19)(q23;p13)		Seq[GRCh37] 46,XX,der(19)t(1;19)(q23;p13.3)		*TCF3::PBX1/TCF3::PBX1*	90%
P119	46,XY,der(19(t(1;19)(q23;p13)		Seq[GRCh37] 46,XY,der(19)t(1;19)(q23;p13)	*TCF3::PBX1*	*TCF3::PBX1/TCF3::PBX1*	90%
P108	46,XX,der(21)qdp(3)del(22.3)	Deletions *EBF1*, *IKZF1*, *CDKN2A*/*B*, *ETV6* and *RB1*	Seq[GRCh37] 46,XXder(21)qdp(21)(q21.1q22.3)del(21)(q22.3)	Deletions *CDKN2A/B*, *EBF1*, and *ETV6* (exon 1) and biallelic deletion *RB1*	iAMP(21)/iAMP(21)	85%
P120	46,XY,t(4;11)(q21;q23).nuc ish(3´KMT2A,5´KMT2A’x2)(3´KMT2A sep5´KMT2Ax1)[28/209]		Seq[GRCh37] 46,XY,t(4;11)(q21;q23.3),inv(13)(q14.11q14.3)	*KMT2A::AFF1*	*KMT2A*-r/*KMT2A*-r	14%
P118	54,XX,+X,+X,+4,t(9;22)(q34;q11),+10,+14,+21,+21,+der(22)t(9;22)		Seq[GRCh37] 54,XX,+X,+X,+4,t(9;22)(q34;q11),+10,+14,+21,+21,+der(22)t(9;22)	*BCR::ABL1* minor	Ph+ ALL/Ph+ ALL	90%
P102	46,XY	Deletions *IKZF1*, *PAX5* and *CDKN2A*	Seq[GRCh37] 46,Y,t(X;14)(p22.33;q32)	*IGH::CRLF2*, *PAX5::ZCCHC7*, and deletion *IKZF1* (exons 4–7)	B-other*/CRLF2*-r and *PAX5*-alt	90%
P103	45,XX,del(7)(p12.2p12.2),dic(9;20)(p13.2;q11.22)	Biallelic deletion *CDKN2A*/*B*, deletions *IKZF1* (exons 4–7) and *PAX5*	Seq[GRCh37] 45,XX,del(7)(p12.2p12.2),dic(9;20)(p13.2;q11.22)	Biallelic deletion *CDKN2A/B*, deletions *IKZF1* (exons 4–6) and *PAX5*	B-other*/PAX5*-alt	95%
P105	46,XX,del(5)(q23.1q34),add(7)(p?1),add(12)(p12),add(12)(p11.2)		Seq[GRCh37] 46,XX,del(5)(q23.1q34),t(7;8;12)(p12.2;q22;p13)	*ETV6::IKZF1*, deletions *CDKN2A/B* and *PAX5* (exons 2–6)	B-other*/ETV6::RUNX1*-like	93%
P106	46,XY,t(12;17)(p13;q12)	Deletions *IKZF1*, *ETV6* and *TP53*	Seq[GRCh37] 46,XY,t(12;17)(p13;q12)	*TAF15::ZNF384* and deletion *IKZF1* (exons 2–6)	B-other*/ZNF384*-r	88%
P107	45,XX,?t(8;10)(p2?;p1),?dic(9;20)(p13;q11)	Biallelic deletion *CDKN2A/B* and deletion *PAX5* (exons 9 and 10)	Seq[GRCH37] 45,XX,der(9;10)(p13;q11.2),dic(9;20)(p13;q11)	*PAX5::SNTA1*, *PAX5::ARHGAP22* and biallelic deletion *CDKN2A/B*	B-other*/PAX5*-alt	86%
P114	46,XY			Deletion *ERG*	B-other*/DUX4*	75%
P115	46,XX,der(1)add(1)(p26.1)add(1)(q3?2),-7,-9,-10,del(12)(p12),-13,add(19)(q13.3),+4mar	Deletions *ETV6*, *IKZF1* and *EBF1*	Seq[GRCh37] 46,XY,t(1;7;2;7)(p13.3;p13.2;q37.1;q36.1),ins(9;2)(p13.2;p11.2p),?der(12)del(12)(p13.2p12.1),der(15)t(12;15)(p13.2;q22.31)	*KMT2C::IKZF1*, deletions *PAX5*, *ETV6*, and *EBF1*	B-other*/ETV6*::*RUNX1*-like and *PAX5*-alt	90%
P117	47,XY,?der(9)del(9)(p21)del(9)(q12q22),-20,+21,+?r		Seq[GRCh37] 46,XY,dic(9;20)(p13.2;q11.2),+21	Biallelic deletion *CDKN2A/B* and deletion *PAX5*	B-other*/PAX5*-alt	90%
P121	45,XY,?dic(4;9)(p?;p13.2),del(14)(q22.1q32.1),der(20)?t(4;20)(p?;q11.2)/45,idem,-8,der(19)t(8;19)(q13;q13),+21	Biallelic deletion CDKN2A/B, deletion IKZF1 (exons 4–7), PAX5 (exons 5–10)	Seq[GRCh37] 45,XY,der(4;20;9)(p14;q11.2;p13.2),del(14)(q22.1q32.11),der(20)t(4;20)(p14;q11.2)/44,idem,-8,der(19)t(8;19)(p21.1;q13.12)	*PAX5::NOL4L*, biallelic deletion, *CDKN2A/B*, deletions *PAX5* (exons 6–10) and *IKZF1* (exons 4–7)	B-other*/PAX5*-alt	98%

The karyotype strings derived from chromosome banding analysis and the WGS-revised strings are written following the International System for Human Cytogenomic Nomenclature (ISCN) 2022 rules (59).

UPN, unique patient number; Seq[GRCh37], Genome Reference Consortium Human Build 37; HeH, high hypodiploidy; HoL, low hypodiploidy; iAMP(21), intrachromosomal amplification of chromosome 21; *KMT2A*-r, *KMT2A* rearrangements; ph+ ALL, Philadelphia-positive ALL; *CRLF2*-r, *CRLF2* rearrangements; *PAX5*-alt, *PAX5* alterations; *ZNF384*-r, *ZNF384* rearrangements; *DUX4*-r, *DUX4* rearrangement.

## Discussion

As the goal of this study was to test the feasibility to replace current multimodal diagnostics with WGS, we assessed the method’s performance to detect all aberrations mandatory in the current treatment protocol for pediatric B-cell ALL patients in the clinical setting. The results show that WGS successfully detected all mandatory events and identified emerging class-defining lesions in the majority of B-other ALL cases. In addition to primary aberrations, the combined analysis of SVs and CNAs enabled the identification of focal and larger losses/gains in all the genes included in the UKALL-CNA classifier (49) and the IKZF+ profile (56). Hence, the very high concordance between WGS findings and SoC results and the excellent diagnostic performance (summarized in **Supplementary Figure 4**) validate our approach for data analysis and interpretation and underscores the utility of WGS as a powerful standalone method in pediatric B-cell ALL diagnostics.

Beyond the established genetic subgroups, the detection of novel genetic lesions in ALL is becoming rapidly relevant, as potentially targetable (13, 16) or lesions affecting outcome have been described (7, 8, 10–16, 51–55). The present approach enabled the identification of at least one potentially class-defining lesion in the majority of B-other ALL (35/39) samples. The most frequently detected lesion was *PAX5*-alt; deletions or amplifications causing haploinsufficiency for *PAX5* were detected in eight of the nine B-other ALL, where *PAX5*-alt was the sole potentially class-defining lesion identified, suggesting that *PAX5* haploinsufficiency might also be a primary lesion. However, PAX5-alt often co-occurred with other class-defining lesions including eight samples with established primary aberrations, which indicates that *PAX5*-alt might also be a secondary alteration. *CRLF2*-r was detected in 10 B-other ALLs and co-occurred with *PAX5* lesions, but never together with any of the established class-defining aberrations. *CRLF2*-r was found as an isolated lesion in the three DS-ALL cases, which is consistent with previous findings that show a high prevalence of *CRLF2*-r among DS-ALL (61). In addition, other recurrent aberrations such as *ZNF384*-r (14), *MEF2D-*r (11), *IGH::ID4* (62), lesions indicating *ETV6::RUNX1-*like ALL such as *ETV6*::*IKZF1* (51), or the very rare *FUS::ERG* fusion (63) were identified among B-other ALL.

While short-read WGS recognized all the mandatory events and many emerging lesions among B-other ALL, no discordant reads linking together *DUX4* gene and IGH locus were found in our initial analysis, neither using the L/N or L-only approach in any of the samples analyzed. Several groups have shown that analysis of global gene expression profiles obtained from RNA-seq is an option to detect *DUX4* rearrangements, as samples harboring *DUX4*-r will cluster together (13, 51, 64). In agreement with these studies, samples P077 and P101 had clustered with *DUX4*-r according to global gene expression analysis, although RNA-seq also failed to detect the *IGH*::*DUX4* fusion transcript (65). Our study included two additional samples suspected to harbor *DUX4*-r indicated by the presence of *ERG* deletion (P050 and P114). We, therefore, searched for *IGH*::*DUX4*-r using a targeted approach focusing on the genomic regions where these genes are located. The approach was successful and identified a high number of discordant reads in support of an *IGH*::*DUX4*-r in these four samples, which was easily discernible from the low signal found among the other samples. Interestingly, the initial SV analysis of the WGS data had revealed an *IGH*-r in P077, and previous examination by 3′RACE had shown a complex rearrangement with intronic sequence from the *CCDC26* locus at 8q24, inserted at the junction between *IGH* and *DUX4*, likely explaining the relatively low number of discordant reads linking together *IGH* and *DUX4* in this particular sample. Thus, we found an *IGH*::*DUX4*-r in 4/39 samples (10%); the discrepancy with other studies that have found a prevalence ranging from 16% to 41% (20, 51) might be due to differences in the bioinformatics approaches or biases in sample selection.

Although WGS represents an unbiased method to interrogate the entire genome, data analysis and interpretation are still challenging. The complexity of the results generated by WGS, partially driven by repetitive genomic elements and benign individual variants, requires effective filtering steps to extract and interpret the relevant findings. This problem was significantly alleviated through annotation using the SweGen reference cohort (43), and the curated in-house database of artifacts and recurrent variants with an observed frequency above 0.02 (44). Furthermore, the use of a short list of genes/regions involved in mandatory and emerging aberrations was instrumental to extract clinically relevant information in an effective manner.

The identification of ploidy changes was straightforward with both the L-only and L/N analyses, and vcf2cytosure correctly identified the expected gains and losses. However, the herein analyzed samples had modal numbers between 53–60 (HeH) and 39 (HoL), implying that the majority of chromosomes were diploid. Potential problems regarding the interpretation of WGS data from L-only may arise for samples if the number of chromosomes is instead close to the haploid genome (n) or multiples thereof, as is the case in near haploid ALL (n±, <30 chromosomes) or near tri/tetraploidy (3n±/4n±, 58–80/81–103 chromosomes). Moreover, the distinction between HeH and duplicated HoL/NH (66) may be challenging using L-only analysis. Nevertheless, as chromosomal gains and losses in these subgroups are not random, the risk of misinterpretation is limited by careful assessment of gains/losses of specific chromosomes (66, 67). These issues can also be solved by including a normal reference as required by ASCAT that enables the detection of CNN-LOH and UPD in addition to aneuploidies.

Most studies have used target sequencing depths ranging from 60× to 90× to assess the diagnostic yield of WGS in hematological malignancies (15, 19, 20, 60). In a study with an effective mean coverage of 50×, Duncavage and coworkers found that the sensitivity was 100% for the detection of CNVs and SVs but decreased to 84.6% for SNVs (19). In the present study, we explored whether decreasing the sequencing depth to 30× could be suitable in the diagnostic setting of ALL and found that 30× could identify all clinically relevant primary aberrations. Moreover, the comparison to SoC demonstrated concordant findings for all cases. Also, a *KMT2A*-r present in only 14% of the cells was confidently detected by targeted analysis of 30× WGS and the L-only approach. These promising findings regarding SV and aneuploidy detection in ALL need, however, further validation in larger studies that include more samples with a low blast count.

In addition to analytical accuracy, the delivery of a timely report to the treating clinician together with cost–benefit aspects is a critical parameter in the implementation of diagnostic tests. In the current ALL trial protocol, the information regarding high-risk genetics and targetable aberrations is required by day 14 at the latest. This precludes using a remission sample or cultured fibroblast DNA from a skin biopsy as the source of constitutional DNA for the L/N analysis in the diagnostic setting. Since the analysis of L-only was equally successful in the identification of all the relevant lesions, we suggest that for diagnostic purposes, this may be the preferred approach. Harmonization and batching with samples investigated for other diseases, e.g., germline conditions, at our hospital were crucial to reduce TAT. Also, filtering the WGS data with a short list significantly simplifies the interpretation task, enabling the delivery of the clinical report within the required TAT. In addition to the time aspect, using L-only will influence the cost–benefit calculations positively, which, together with the steady drop in sequencing costs over the past years, may render replacing the current multimodal SoC with WGS a realistic option (18, 68). The micro-costing aspects of replacing SoC with WGS are addressed in an ongoing prospective study of patients diagnosed with acute leukemia (69); however, a preliminary estimation indicates that assuming that WGS can replace the current multimodal SoC testing, the cost per patient will increase with a factor of roughly 1.2 (data not shown).

Increasing the clinical benefit of WGS will also contribute to driving the cost–benefit balance in the desired direction. While the present study was designed to validate the diagnostic accuracy of somatic lesions relevant for ALL stratification, the data generated by WGS can be further used for other diagnostic purposes. In ALL, the early response to therapy is the most important prognostic factor, and thus, monitoring response is critical to managing the individual patient. In a proof-of-principle study, WGS has been shown to enable the identification of patient-specific unique sequences that constitute highly specific and sensitive markers that yielded quantitative assays whose performance can potentially outperform SoC (70). Moreover, the genomic data provided by WGS can also be used to extract pharmacogenetic information regarding variants that affect drug metabolism or to investigate the presence of germline variants, information that may contribute additional valuable input to patient management (7).

In summary, we identified all samples with ploidy changes and called the individual trisomies/tetrasomies or monosomies correctly. Moreover, we detected the SVs that lead to oncogenic gene fusions or iAMP (21) and were able to determine CNA profiles. The WGS results generated showed excellent concordance with SoC findings and allowed allocation to the correct genetic subgroups in all cases. In addition, WGS detected lesions not routinely investigated in SoC, and consequently, we were able to identify primary class-defining aberrations in the majority of B-other ALL samples including *DUX4*-r and to allocate the samples to one of the emerging genetic subgroups. We conclude that our strategy was successful in extracting clinically relevant information from paired-end WGS and that the analysis of L-only detected all clinically relevant aberrations with the same accuracy as paired analysis. Hence, WGS as the sole method represents an accurate and promising diagnostic tool in ALL diagnostics.

## Data availability statement

The data presented in the study are deposited in the https://figshare.scilifelab.se repository, accession number 23530605.

## Ethics statement

The studies involving human participants were reviewed and approved by the Ethical Review Board at Stockholm County or the Uppsala County. Written informed consent to participate in this study was provided by the participants’ legal guardian/next of kin.

## Author contributions

GB, LF, RR, LC, BJ, LP, TF, and VW designed the study and secured the funding. JE, AS, HL, and KM performed the bioinformatics analysis. GB, JE, and FR performed the data acquisition, analysis, and interpretation, and IÖ contributed to data analysis and interpretation. JN, ACS, and SS contributed to the data acquisition, and IÖ, LO-A, AS, and COP contributed to the data interpretation. FR and GB wrote the manuscript. LF, JN, IÖ, and RR contributed to the writing of the manuscript. All authors revised and approved the final version of the manuscript.

## Supplementary material

Supplementary Figure 1 Visualization of aneuploidies and large CNAs. Screenshots from vcf2cytosure (upper panel) and ASCAT (lower panel) illustrating the results for a representative sample with, **(A)** HeH (P029) showing the signal intensity across all chromosomes. **(B)** iAMP (21) showing the signal intensity along chromosome 21 (P075). The red line indicates the signal intensity corresponding to diploid chromosomes, signals above this threshold indicate gain and below losses.

Supplementary Figure 2 Visualization of focal CNAs. IGV screenshots from representative samples illustrating representative CNAs. IGV screenshots show the discordant reads at both ends of the junction displayed in colored bars and concordant reads displayed in grey. showing **(A)** *IKZF1* deletion of exons 4-7 (P049), **(B)** amplification of *PAX5* exons 2-5 (P060), **(C)** *ERG* deletion (P050), **(D)** PAR1 deletion (P049) resulting in the fusion *CRLF2::P2RY8*.

Supplementary Figure 3 Illustration of representative SVs in B-other group. Screenshots from IGV and Circos plot illustrating representative B-other aberrations. IGV screenshots show the discordant reads at both ends of the junction displayed in colored bars and concordant reads displayed in grey. **(A)** dic(9;20) (P042), **(B)** *IGH::CRLF2* t(X;14) (P057), **(C)** *TCF3::ZNF384* t(12;19) (P052), **(D)** a rare fusion gene found in one case *FUS::ERG* t(16;21) (P089).

Supplementary Figure 4 Comparison SoC and WGS. Chart showing which SoC methods can be used to detect the primary class-defining lesions in ALL or to call CNA profiles. The brackets indicate that some but not all lesions included in the subgroup can be detected by the method. The asterisk denotes subgroups defined by global gene expression analysis.

Supplementary Table 1 Mandatory aberrations in ALLtogether treatment protocol.

Supplementary Table 2 Short list of clinically relevant genes/genomic regions in ALL Chr., chromosome; HGNC ID, HUGO Gene Nomenclature committee ID.

Supplementary Table 3 Genomic findings in samples harboring mandatory aberrations. UPN, unique patient number; HeH, high hyperdiploidy; iAMP (21), intrachromosomal amplification of chromosome 21;*KMT2A*-r, *KMT2A* rearrangements; SoC, standard of care; seq[GRCh37], Genome Reference Consortium Human Build 37; UPD, uniparental disomy; CNN-LoH, Copy number neutral- loss of heterozygosity. The karyotype strings derived from chromosome banding analysis and the WGS-revised strings are written following the International Standards for Cytogenetic Nomenclature, ISCN 2022 rules (59).

Supplementary Table 4 Summary of SoC and WGS findings in B-other group. UPN, unique patient number; Seq[GRCh37], Genome Reference Consortium Human Build 37; CNN-LoH, Copy number neutral- loss of heterozygosity; *PAX5*-alt, *PAX5* alterations; *CRLF2*-r, *CRLF2* rearrangements; *ZFN384*-r, *ZFN384* rearrangements; *IGH*-r, *IGH* rearrangement; *DUX4*-r, *DUX4* rearrangements. The karyotype strings derived from chromosome banding analysis and the WGS-revised strings are written following the International Standards for Cytogenetic Nomenclature, ISCN 2022 rules (59).

Supplementary Table 5 Number of reads supporting an *IGH*::*DUX4* rearrangement.

Supplementary information SAMtools command to count the reads supporting *IGH*::*DUX4*-r.
