# Supplementary figures and images for: Feasibility to use whole-genome sequencing as a sole diagnostic method to detect genomic aberrations in pediatric B-cell acute lymphoblastic leukemia
Source: Front Oncol. 2023 Aug 14;13:1217712. doi: 10.3389/fonc.2023.1217712 (PMC10470829; doi:10.3389/fonc.2023.1217712)

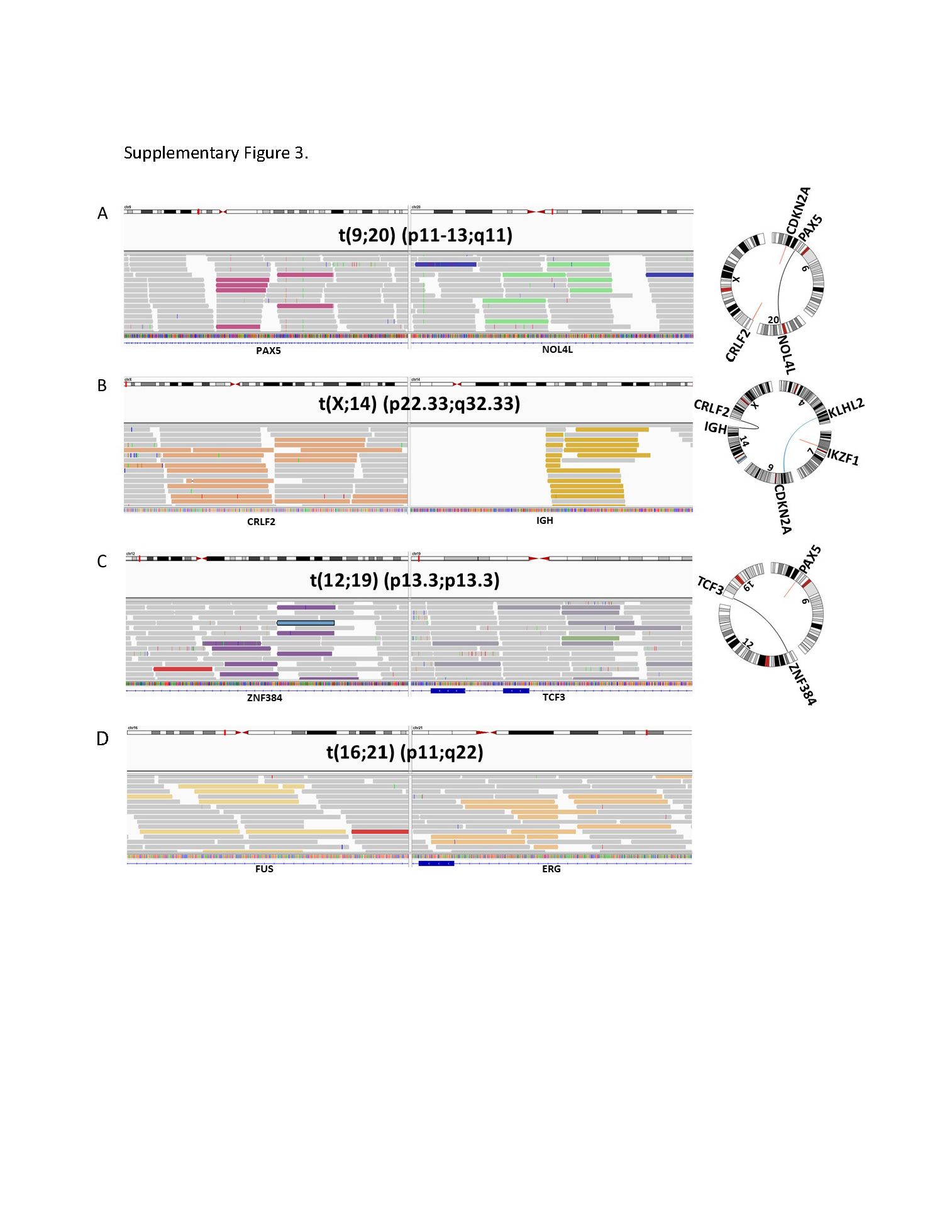

Supplement: Supplementary Figure 1 — Visualization of aneuploidies and large CNAs. Screenshots from vcf2cytosure (upper panel) and ASCAT (lower panel) illustrating the results for a representative sample with, (A) HeH (P029) showing the signal intensity across all chromosomes. (B) iAMP (21) showing the signal intensity along chromosome 21 (P075). The red line indicates the signal intensity corresponding to diploid chromosomes, signals above this threshold indicate gain and below losses. [file Image_1.tif]

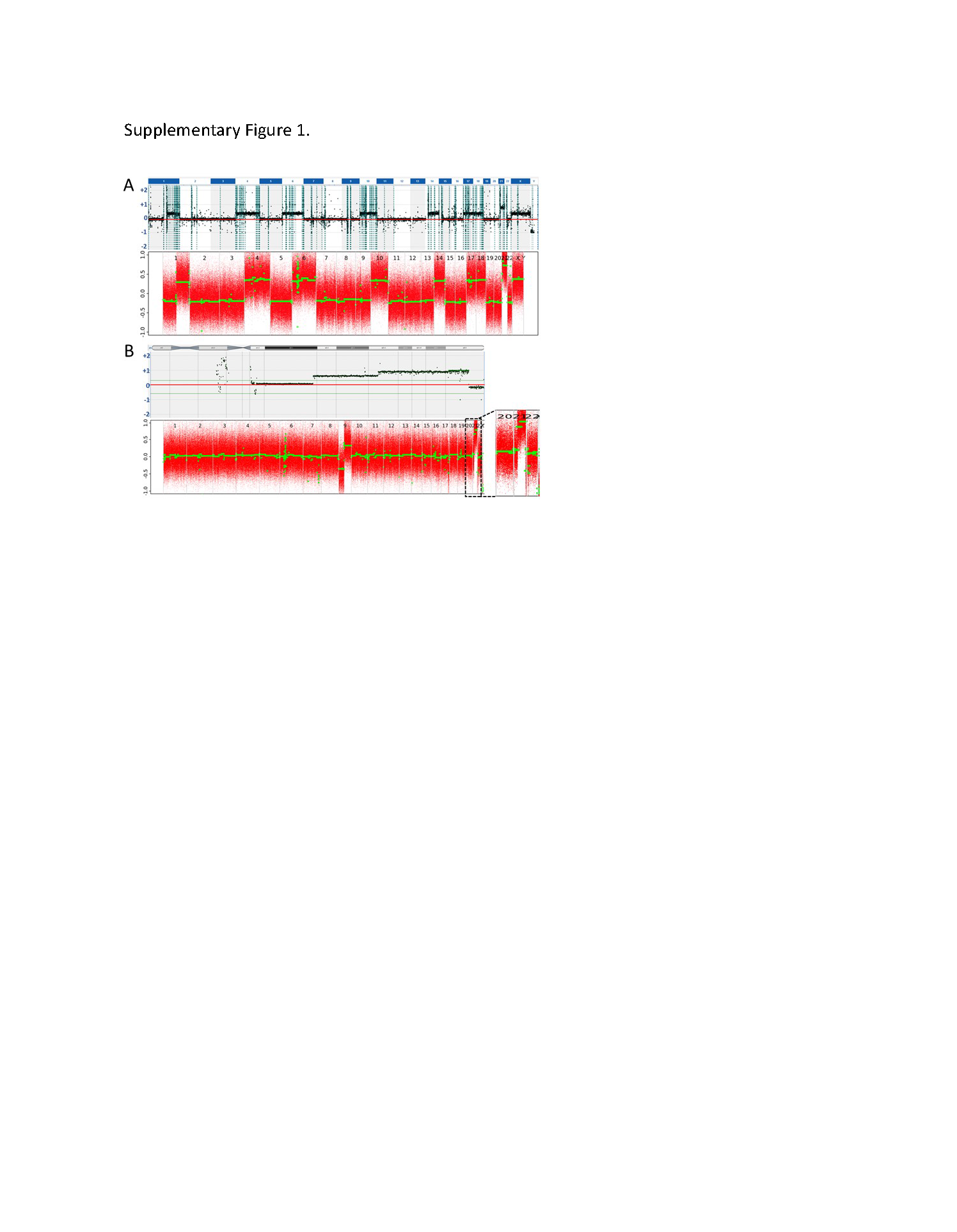

Supplement: Supplementary Figure 2 — Visualization of focal CNAs. IGV screenshots from representative samples illustrating representative CNAs. IGV screenshots show the discordant reads at both ends of the junction displayed in colored bars and concordant reads displayed in grey. showing (A) IKZF1 deletion of exons 4-7 (P049), (B) amplification of PAX5 exons 2-5 (P060), (C) ERG deletion (P050), (D) PAR1 deletion (P049) resulting in the fusion CRLF2::P2RY8. [file Image_2.tiff]

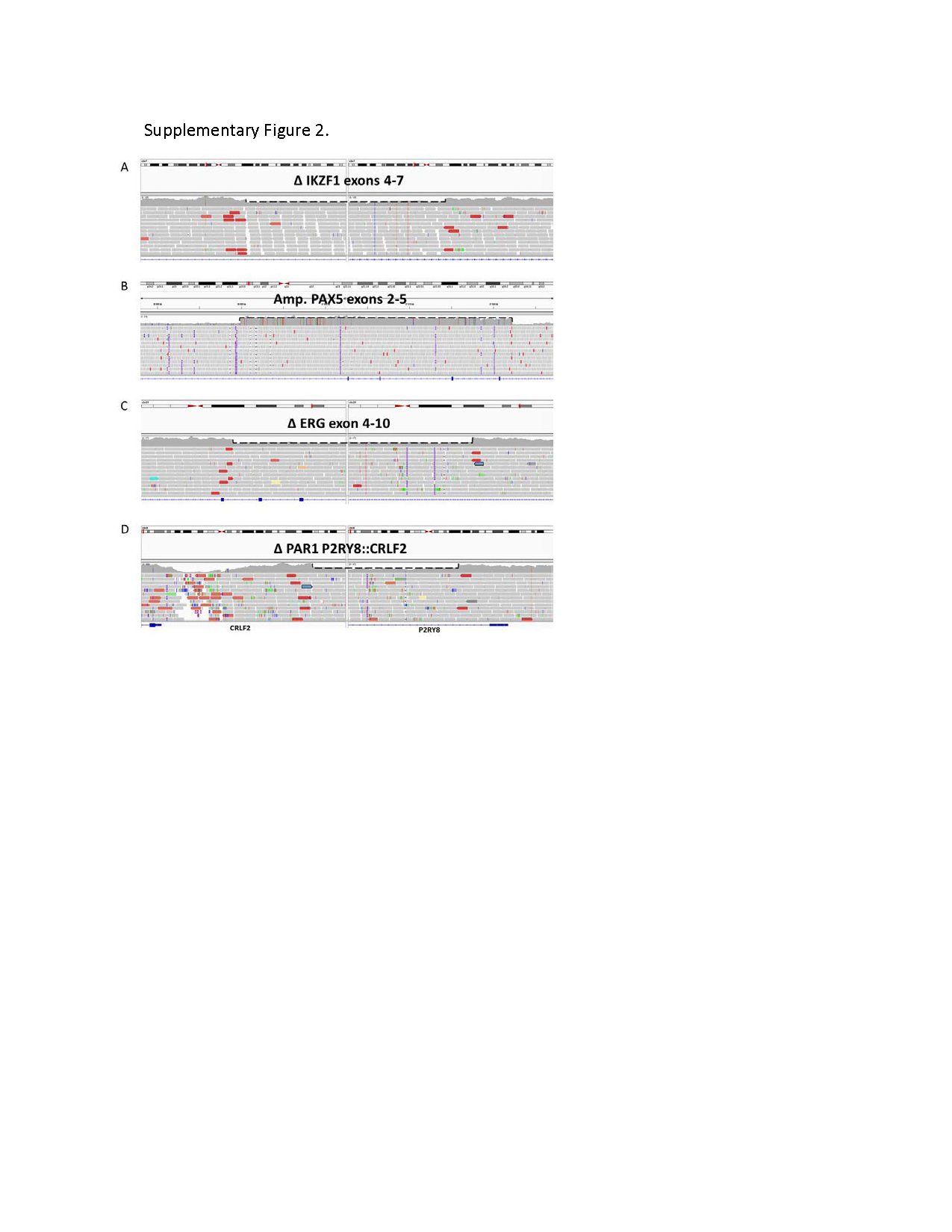

Supplement: Supplementary Figure 3 — Illustration of representative SVs in B-other group. Screenshots from IGV and Circos plot illustrating representative B-other aberrations. IGV screenshots show the discordant reads at both ends of the junction displayed in colored bars and concordant reads displayed in grey. (A) dic(9;20) (P042), (B) IGH::CRLF2 t(X;14) (P057), (C) TCF3::ZNF384 t(12;19) (P052), (D) a rare fusion gene found in one case FUS::ERG t(16;21) (P089). [file Image_3.tiff]

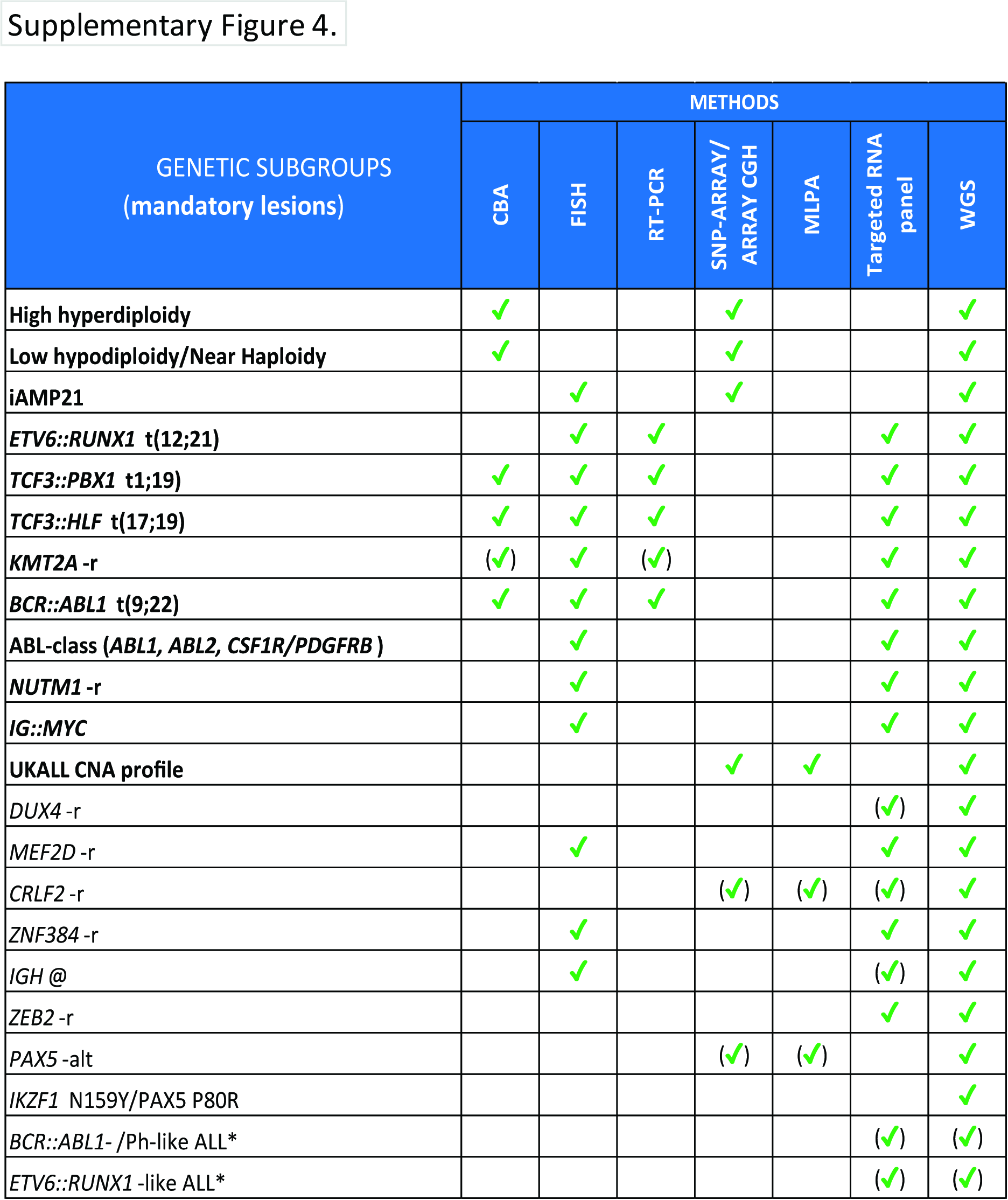

Supplement: Supplementary Figure 4 — Comparison SoC and WGS. Chart showing which SoC methods can be used to detect the primary class-defining lesions in ALL or to call CNA profiles. The brackets indicate that some but not all lesions included in the subgroup can be detected by the method. The asterisk denotes subgroups defined by global gene expression analysis. [file Image_4.tif]
