# Supplementary material for: Feasibility to use whole-genome sequencing as a sole diagnostic method to detect genomic aberrations in pediatric B-cell acute lymphoblastic leukemia
Source: Front Oncol. 2023 Aug 14;13:1217712. doi: 10.3389/fonc.2023.1217712 (PMC10470829; doi:10.3389/fonc.2023.1217712)
Supplement: Supplementary information — SAMtools command to count the reads supporting IGH::DUX4-r. [file DataSheet_1.docx]

**Supplementary information**

The following SAMtools command was issued to identify reads indicating the presence of an *IGH::DUX4* rearrangement, based on the presence of D4Z4 repeat region in the reference genome used for alignment:

samtools view -F 1024 -c -e '(rnext == "4" && pnext > 190988100 && pnext < 191007000) || (rnext == "10" && pnext > 135477000 && pnext < 135500000) || (rnext == "GL000228.1" && pnext > 70000 && pnext < 115000) || ([SA] =~ "10,1354[789][0-9]{4}") || ([SA] =~ "4,19(09[8-9][0-9]|100[0-7])[0-9]{3}" || [SA] =~ "GL000228.1,([7-9][0-9]{4}|1[0-1][0-5][0-9]{3})")' WGS.bam 14:106032614-107288051

The flag -F 1024 removes PCR duplicates. The flag -c tells samtools to only report the number of reads matching this command, instead of writing out the mapped read information.
